# Influence of mindfulness and coping flexibility in the early phases of burnout development in intensive care unit healthcare workers during the COVID-19 pandemic

**DOI:** 10.1371/journal.pone.0328064

**Published:** 2025-08-21

**Authors:** Damien Claverie, Anaïs Duffaud, Sonia Pellissier, Sandrine Jacob, Cécile Vigier, Marc Danguy des Déserts, Pierre Pasquier, Jacques Escarment, Frédéric Canini, Marion Trousselard

**Affiliations:** 1 Unité Neurophysiologie du Stress, Département Neurosciences et Contraintes Opérationnelles, Institut de Recherche Biomédicale des Armées, Brétigny-sur-Orge, France; 2 Université Savoie Mont Blanc, Université Grenoble Alpes, Laboratoire Interuniversitaire de Psychologie, Personnalité, Cognition, Changement Social (LIP/PC2S), Grenoble, France; 3 Réseau ABC des Psychotraumas, Montpellier, France; 4 Pôle Bloc Anesthésie, Réanimation, Urgences, Hôpital Régional d’Instruction des Armées Clermont-Tonnerre, Brest, France; 5 Université Brest, Inserm, UMR 1304-GETBO, Brest, France; 6 École du Val-de-Grâce, Paris, France; 7 Anesthésie-réanimation, Hôpital National d’Instruction des Armées Percy, Clamart, France; 8 Bureau des Officiers Généraux de la Direction Centrale du Service de Santé des Armées, Paris, France; 9 APEMAC, EA 4360, Université de Lorraine, Nancy, France; Shahrood University of Medical Sciences, IRAN, ISLAMIC REPUBLIC OF

## Abstract

**Background:**

Caregivers in intensive care units are exposed to high levels of stress, which were exacerbated during the COVID-19 pandemic, increasing the incidence of stress-related disorders, including burnout.

**Methods:**

We evaluated the influence of mindfulness and coping flexibility in the early days of burnout development in a prospective study of 50 military caregivers working in a mobile resuscitation unit during the COVID-19 pandemic. We visited the participants at the end of deployment (day 0; D0) and three weeks later (D21). On D0, the participants completed questionnaires assessing mindfulness (Freiburg Mindfulness Inventory; FMI), coping flexibility (Flexcop), and burnout (Burnout Measure, Short Version; BMS). Subjective sleep symptoms were also assessed to evaluate their relationship with burnout. The BMS was repeated on D21.

**Results:**

Four short-term burnout evolutions were observed: (i) “healthy” on D0 and D21; (ii) “exhausted,” healthy on D0 and burnout on D21; (iii) “resilient,” burnout on D0 and healthy on D21; and (iv) “burnout” on D0 and D21. Compared with healthy participants on D0, only resilient participants had lower FMI (η2 = 0.22; p = 0.032). Exhausted participants had more difficulty waking, and burnout participants had higher daytime fatigue than healthy participants. FMI scores on D0 negatively correlated with BMS scores on D21 (r = ‒0.40 r^2^ = 0.16 p = 0.009) for the entire population, and also after excluding burnout status at D0 (r = ‒0.41 r^2^ = 0.17 p = 0.02). Flexcop scores on D0 did not correlate with BMS scores on D21.

**Conclusions:**

This study underlines the importance of mindfulness in the early days of burnout onset, and suggests considering its reinforcement for prevention of burnout and its measurement as a potential biomarker of burnout risk.

## Introduction

The COVID-19 pandemic increased psychological burden in caregivers [1], especially those working in intensive care units (ICUs) [2]. Burnout is an important psychopathology in caregivers working in ICUs [3] that usually accompanies work-related wear [4]. Burnout is associated with high stress levels [5] and poor sleep quality [6]. A common feature of burnout is emotional exhaustion [7]. Unfortunately, few longitudinal studies have been conducted on burnout in caregivers working in ICUs under the work pressures of the COVID-19 pandemic [8]. Caregivers working in units where employees are more prone to have work-related stress may be protected from ill-health by mindfulness [9].

Mindfulness is one of the potential protective factors of burnout; mindfulness disposition is the ability to focus on the present (presence dimension) with an attitude of acceptance in everyday life (acceptance dimension) [10]. Presence and acceptance favour psychological flexibility that might moderate different adaptive skills [11]. Mindful individuals may experience less psychological distress and, subsequently, a reduced risk of emotional exhaustion [12]. The restorative role of mindfulness may protect against depersonalisation by focusing attention on the present [13]. Mindfulness is positively associated with emotional regulation and a low stress reaction in response to a challenge [14]. Improved emotional regulation could explain the benefit of mindfulness-based interventions for burnout prevention [14], leading to the hypothesis that being mindful could be a protective factor against burnout.

Coping flexibility, using different ways of coping with a stressful environment, helps caregivers deal with stressors to reduce stress reactions [15,16]. This approach depends on two processes: (i) the individual’s ability to determine coping effectiveness and (ii) the ability to change the type of coping [16]. Because burnout does not seem to be linked with the type of coping strategies used [17], the role of coping flexibility to predict burnout remains to be explored. Indeed, a lack of coping flexibility might increase emotional load due to a non-adjusted coping strategy [18]. We hypothesise that having a high coping flexibility protects against burnout.

We conducted a prospective study that carried out in a working environment focusing on a caregiver population in the specific context of a field deployment in a military intensive care hospital during the COVID-19 pandemic. The study had three aims: (i) to quantify burnout in the early phases in caregivers working in an ICU during the COVID-19 pandemic using the Burnout Measure, Short Version (BMS); (ii) to evaluate the role of mindfulness and coping flexibility in the onset of burnout; and (iii) to determine the psychophysiological correlates of the BMS by assessing the activity of the autonomic nervous system activity using electrocardiogram (ECG) and electrodermal activity (EDA) data and by quantifying subjective sleep quality [19].

## Materials and methods

### 1. Population

**Fig 1:**
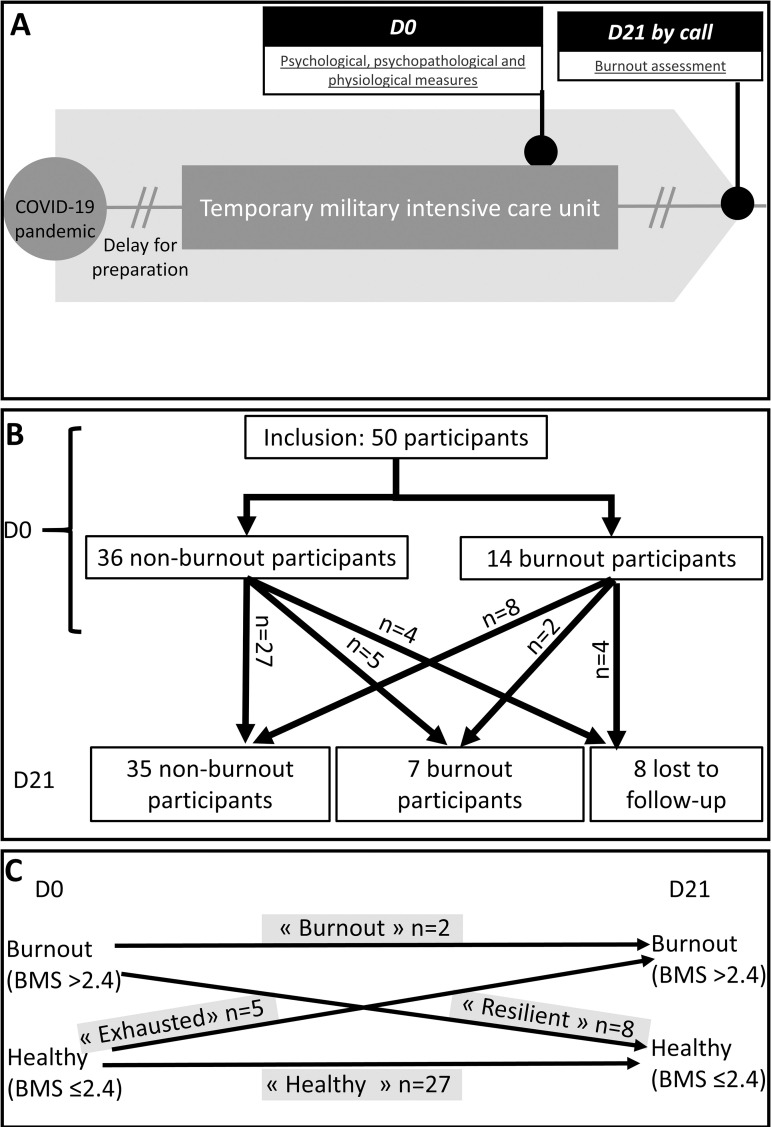
Study protocol and flowchart. **A.** Study protocol. **B.** Flowchart of the study in accordance with the burnout state. **C.** Psychopathological pathways of the four groups, highlighted in grey (healthy, exhausted, resilient, and burnout). Burnout was defined as a BMS score greater of 2.4. BMS, Burnout Measure, Short Version; D, day.

The study was an observational study conducted on 50 military caregivers deployed for 30–60 days to a field military intensive care hospital in Mulhouse, eastern France, at the peak of the first wave of COVID-19 infection in early 2020 (Fig 1A). The population size was calculated based on data from the FMI questionnaire in a previously published study of a military population (including military caregivers) conducted prior to an operational deployment [20]. The average population score was approximately 39, with a standard deviation of 6.89. As a difference of 20% (7 in absolute value) in this score would be considered clinically significant, and with a fixed type I error rate of 5% and a fixed power of 90% in a two-tailed hypothesis, the required sample size for the study was determined to be 42 patients. This number was rounded up to 50 to account for potential attrition, which is commonly described as being at 20%. It is worth noting that this population represented almost half of the staff at the “temporary hospital” deployed for the pandemic, which reinforces the relevance of our results. Recruitment was carried out through informational posters in the living area. Recruitment stopped once the number of subjects included reached 50. The recruitment (D0) started on April 25^th^, 2020, and ended on April 27^th^, 2020, which corresponds to the week before the end of the deployment for these personnel. Note that the caregivers had been working in the COVID-19 pandemic context at their respective hospitals, and then at this temporary hospital, for approximately two months before this recruitment. Other studies were previously published in this same context [21–23]. The study ended 21 ± 7 days after the end of deployment (D21).

The inclusion criteria were to be a volunteer staff member of the Mulhouse mobile intensive care unit, including military reservists. The exclusion criteria were being a pregnant or breastfeeding woman, being deprived of liberty by a judicial or administrative decision, being subject to a legal protection measure or unable to give consent, having an intercurrent pathology with inability to work, or having a history of psychiatric disorder or cardiac pathology. The study was approved by the French Military Medical Service ethics committee and the French Health Authorities (identification number 2020-A01058.31) and was declared on ClinicalTrial.gov (NCT04365335). Written informed consent was obtained from all participants in conformity with the Declaration of Helsinki.

### 2. Data collection tools and procedures

Data were collected in two sessions (Fig 1A): (i) the first session on day 0 (D0) took place 7 ± 4 days before the end of deployment and (ii) the second session took place 21 ± 7 days after the end of deployment (D21). Participants were asked not to drink coffee, or to smoke, 1 hour before the first session on D0. On D0, participants were given 30 minutes to complete written standardised assessments that collected common sociodemographic data and evaluated psychopathological (burnout, post-traumatic stress disorder (PTSD), depression, and anxiety), psychological (mindfulness and coping flexibility), and physiological (sleep) symptoms. ECG and EDA data were recorded from the participants during a 20-minute emotional exposure protocol (EEP). Participants were called by phone on D21 to repeat the BMS questionnaire.

The participants were categorised into four groups in accordance with their BMS status (burnout vs. non burnout) on D0 and D21 (i.e., healthy, exhausted, resilient, and burnout participants; Fig 1B, 1C).

### 3. Psychopathological evaluation

#### a. BMS.

The BMS questionnaire evaluates the amount of burnout as a unidimensional construct with high face validity [24] and good stability over time [25]. The BMS ranges from 1 to 7. It identifies four levels of burnout severity: light burnout (>2.4 to ≤3.4), burnout (>3.4 to ≤4.4), severe burnout (>4.4 to ≤5.4), and very severe burnout (>5.4) [25]. BMS scores were evaluated on D0 and D21, and changes to burnout levels were calculated as BMS on D21 − BMS on D0.

#### b. Hospital Anxiety and Depression Scale (HADS).

The HADS aims to detect anxiety and depression in non-psychiatric medical outpatients [26]. The HADS ranges from 0 to 40 for anxiety and for depression subscales. The score increases according to the severity. We chose a cut-off of ≥11 for anxiety and for depression to favour specificity (0.92) over sensitivity (0.56) [27]. The HADS questionnaire was administered on D0.

#### c. Post-Traumatic Stress Disorder Checklist (PCL-5).

The PCL-5 was used to detect PTSD on D0 [28]. The PCL-5 ranges from 0 to 80. The score increases according to the severity of PTSD symptoms. We chose a cut-off of ≥33, as validated in a French population [29].

### 4. Psychological evaluation on D0

#### a. Freiburg Mindfulness Inventory (FMI).

The FMI scale measures the two dimensions of mindfulness: presence and acceptance. It consists of 14 items, each rated from 1 to 5. Accordingly, the score varies from 14 to 70. The higher the score, the more mindful the participant.

#### b. Coping Flexibility Scale (Flexcop).

The Flexcop, evaluates coping flexibility (metacoping) in two dimensions: evaluation (the ability to evaluate the effectiveness of the coping used) and adaptation (the ability to change the type of coping when it has been judged to be inadequate) [30]. It is a 10-item Likert-based scale ranging from 1 to 4. The score ranges from 10 to 40 with higher values indicating greater coping flexibility [31].

### 5. Physiological evaluation on D0

#### a. Leeds Sleep Evaluation Questionnaire (LSEQ).

The LSEQ evaluates four aspects of sleep: (i) sleep quality, (ii) wake quality, (iii) awakening quality, and (iv) performance [32].

### 6. EEP

#### a. Protocol.

The EEP was carried out on D0. Each participant was seated comfortably throughout the protocol. The EEP consisted of four, 5-minute phases: (i) resting baseline, during which the participants were asked to keep quiet and to not move while in a seated position; (ii) emotional exposure, during which the participants were asked to recall a recent intense emotionally negative event experienced during deployment; (iii) questionnaire, during which the participants completed the Perceived Stress Scale (PSS) questionnaire; and (iv) relaxation.

#### b. Electrophysiological recordings.

The ECG and EDA analyses were carried out for the whole of the 20-minute EEP session. We used the ECG data to calculate the root mean square of successive differences (RMSSD; in ms), very low frequencies (VLF), low frequencies (LF), and high frequencies (HF). The EDA data were also used to calculate the mean tonic EDA and the height and number of phasic EDA (S1 File). These signals were further analysed in another study focusing on electrophysiology.

#### c. PSS.

The PSS assesses the degree to which the respondent perceives situations as stressful [33]. The PSS is a self-report scale of 14 items with a score from 0 to 56 with higher values indicating greater perceived stress. The questionnaire was given during the EEP to assess perceived stress when remembering events that occurred during deployment.

### 7. Statistical analysis

All statistical analyses were performed using Statistica version 7.1 (StatSoft, USA) and MATLAB R2019a (MathWorks, USA). Normality was verified with a Kolmogorov-Smirnov test. If normality was verified (p > 0.05), comparisons among groups were carried out using factorial analysis of variance tests followed, if necessary, by *post-hoc* Dunnett tests using the healthy group data for comparisons. If normality was not verified, comparisons among group were carried out using a non-parametric Kruskal-Wallis test followed, if necessary, by post-hoc multiple comparison test. Categorical comparisons among groups were carried out using χ^2^ tests. The statistical threshold of significance was set at p < 0.05, and statistical trends were set at p < 0.10. Data are expressed as mean ± SEM. Correlations were calculated using Pearson regression methods, and we considered clinically significant only results for which r^2^ ≥ 0.10. To assess links between variables we performed factorial analyses based on a normalised varimax rotation [34].

## Results

### 1. Characterisation of burnout on day 0

#### a. Burnout vs. non-burnout.

At the start of the study (D0), the 50 participants included in the study were aged between 19 and 64 years. 14 participants presented with burnout (13 with light burnout, 2.4 < BMS ≤ 3.4; 1 with severe burnout, BMS = 4.6) whereas 36 participants did not. The participants with burnout had higher HADS-Depression (p = 0.003 η2 = 0.18; Table 1A), a tendency for higher PCL-5 (p = 0.06 η2 = 0.07; Table 1A), and no differences in HADS-Anxiety (p = 0.11 η2 = 0.05) than non-burnout participants, but the number of participants that were anxious, depressed, or had PTSD did not differ significantly between the burnout and non-burnout groups (S1 Table).

**Table 1:** Characterisation of burnout in participants on D0. A. Psychopathological parameters of burnout. Results are expressed as mean ± SEM. Results with p < 0.10 for analysis of variance are highlighted in bold. B. Factorial analysis of studied variables on day 0. Factorial weights are displayed. Factorial weights with absolute value higher than 0.50 are in bold. C. Pearson correlation data between D0 BMS scores and the factorial analysis’s factors and variables. r^2^ higher or equal to 0.10 are in bold. BMS, Burnout Measure, Short Version; D, day; Flexcop, coping flexibility; FMI, Freiburg Mindfulness Inventory; HADS, Hospital Anxiety and Depression Scale; PCL-5, Post-Traumatic Stress Disorder Checklist for DSM-5; PSS, Perceived Stress Scale.

**A.**			
	Non-burnout	Burnout	Statistics
HADS-Anxiety	5.74 ± 0.51	7.30 ± 0.78	F(1,47)=2.67 p = 0.11 η2 = 0.05
HADS-Depression	2.03 ± 0.31	4.38 ± 0.90	**F(1,47)=10.08 p = 0.003 η2 = 0.18**
PCL-5	9.55 ± 1.52	15.23 ± 2.45	**F(1,47)=3.77 p = 0.058 η2 = 0.07**
**B.**			
	Factor 1	Factor 2	Factor 3
FMI	−0.12	−0.02	**−0.79**
Flexcop	−0.07	0.07	**−0.84**
PSS	0.28	0.13	**0.57**
Difficulty falling asleep compared to usual	0.32	**0.80**	0.13
Duration to falling asleep compared to usual	0.13	**0.84**	0.13
To be sleepy compared to usual	0.20	**−0.79**	0.07
Restful sleep compared to usual	**−0.59**	**−0.66**	−0.02
Fragmented sleep compared to usual	**0.63**	**0.55**	−0.13
Easy awakening compared to usual	**−0.80**	0.03	−0.16
Fast awakening compared to usual	**−0.80**	0.02	−0.12
To be tired compared to usual	**0.82**	0.14	0.36
Daytime fatigue compared to usual	**0.55**	0.16	0.38
Disturbed sleep compared to usual	**0.74**	0.27	0.13
**Eigenvalue**	3.79	2.84	2.04
**C.**			
	D0 BMS Pearson correlations		
Factor 1	r = 0.28 r^2^ = 0.08 p = 0.053		
Factor 2	r = 0.03 r^2^ = 0.0009 p = 0.83		
**Factor 3**	**r = 0.40 r** ^2^ ** = 0.16 p = 0.004**		
FMI Presence	r=−0.21 r^2^ = 0.04 p = 0.15		
**FMI Acceptance**	**r=−0.39 r** ^2^ ** = 0.16 p = 0.006**		
**FMI total**	**r=−0.37 r** ^2^ ** = 0.14 p = 0.009**		
Flexcop Evaluation	r=−0.26 r^2^ = 0.07 p = 0.18		
Flexcop Adaptation	r=−0.26 r^2^ = 0.07 p = 0.13		
Flexcop total	r=−0.27 r^2^ = 0.07 p = 0.06		
**PSS**	**r = 0.37 r** ^2^ ** = 0.14 p = 0.009**		
Difficulty falling asleep compared to usual	r = 0.19 r^2^ = 0.04 p = 0.18		
Time taken to fall asleep compared to usual	r = 0.20 r^2^ = 0.04 p = 0.16		
Sleepiness compared to usual	r = 0.17 r^2^ = 0.03 p = 0.23		
Restful sleep compared to usual	r = 0.01 r^2^ = 0.0001 p = 0.95		
Fragmented sleep compared to usual	r = 0.11 r^2^ = 0.01 p = 0.43		
Easy awakening compared to usual	r=−0.19 r^2^ = 0.04 p = 0.18		
Fast awakening compared to usual	r=−0.24 r^2^ = 0.06 p = 0.10		
**Tiredness compared to usual**	**r = 0.40 r** ^2^ ** = 0.16 p = 0.004**		
Daytime fatigue compared to usual	r = 0.29 r^2^ = 0.08 p = 0.04		
**Disturbed sleep compared to usual**	**r = 0.33 r** ^2^ ** = 0.11 p = 0.02**		

#### b. Associations between burnout and psychological variables.

To explore the hidden latent factors, a factorial analysis was performed on all studied variables based on a normalised varimax rotation (Table 1B). Three factors were observed: Factor 1, sleep and awakening qualities; Factor 2, falling asleep and sleep qualities; and Factor 3, psychological functioning.

The association between the BMS and other variables was determined by Pearson correlation analysis (Table 1C). The BMS was positively correlated with Factor 3, psychological functioning (r = 0.40; r^2^ = 0.16 p = 0.004). We also observed correlations of the BMS with the FMI and the FMI-Acceptance (r=−0.39; r^2^ = 0.16 p = 0.006 and r=−0.37; r^2^ = 0.14 p = 0.009) and the PSS (r = 0.37; r^2^ = 0.14 p = 0.009) but not with Flexcop (r=−0.27; r^2^ = 0.07 p = 0.06; Table 1C). Sleep symptoms (being tired (r = 0.40; r^2^ = 0.16 p < 0.01) and having disturbed sleep (r = 0.33; r^2^ = 0.11 p = 0.02)) also positively correlated with BMS.

### 2. Burnout changes from D0 to D21

#### a. Burnout changes.

**Fig 2:**
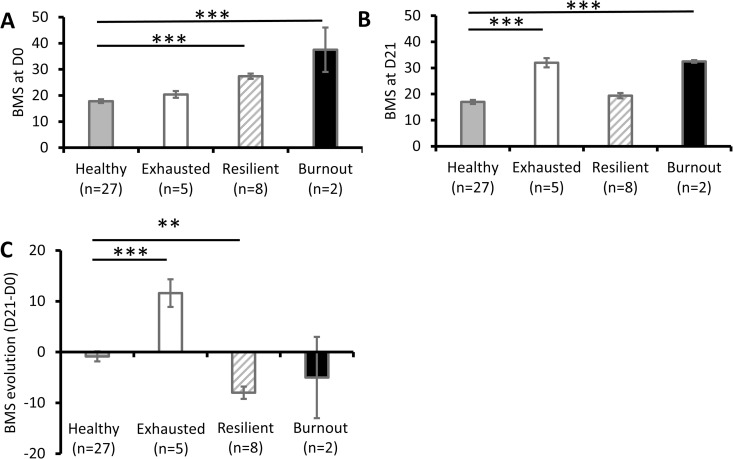
Burnout changes in participant groups. **A.** BMS scores on D0. **B.** BMS scores on D21. **C.** BMS score changes from D0 to D21. Results are expressed as mean ± SEM. **: p < 0.01, ***: p < 0.001; based on post-hoc tests. BMS, Burnout Measure, Short Version; D, day.

We characterised the groups defined by changes in BMS from D0 to D21 (Fig 1B). (i) Healthy participants had low BMS on D0 and D21 (Fig 2A–2C). All comparisons were made with healthy group. (ii) exhausted participants had higher BMS on D21 (F(3,41)=30.28, p = 3.58x10^-10^ η2 = 0.71; *post-hoc*, p = 8.47x10^-6^; Fig 2B) and higher BMS changes from D0 to D21 (F(3,41)=15.06, p = 1.29x10^-6^ η2 = 0.54; *post-hoc*, p = 5.86x10^-5^; Fig 2C); (iii) resilient participants had higher BMS on D0 (F(3,41)=24.93, p = 4.36x10^-9^ η2 = 0.66; *post-hoc*, p = 9.83x10^-6^; Fig 2A) and lower BMS changes from D0 to D21 (F(3,41)=15.06, p = 1.29x10^-6^ η2 = 0.54; *post-hoc*, p = 0.005; Fig 2C); and (iv) burnout participants had higher BMS on D0 (F(3,41)=24.93, p = 4.36x10^-9^ η2 = 0.66; *post-hoc*, p = 8.58x10^-6^; Fig 2A) and D21 (F(3,41)=30.28, p = 3.58x10^-10^ η2 = 0.71; *post-hoc*, p = 1.38x10^-5^; Fig 2B).

#### b. Sociodemographic and psychopathological characterisation.

No differences were observed between the healthy, exhausted, resilient, and burnout groups for sociodemographic characteristics (S2 Table), psychopathology repartitions (S2 Table), or psychopathology scores (HADS-Depression (F(3,37)=2.22, p = 0.102 η2 = 0.15; S1 Fig), HADS-Anxiety (F(3,37)=2.28, p = 0.096 η2 = 0.16; *post-hoc,* not significant) or PCL-5 (F(3,37)=1.83, p = 0.16 η2 = 0.13) (S1 Fig)) on D0.

#### c. Psychological characterisation.

**Fig 3:**
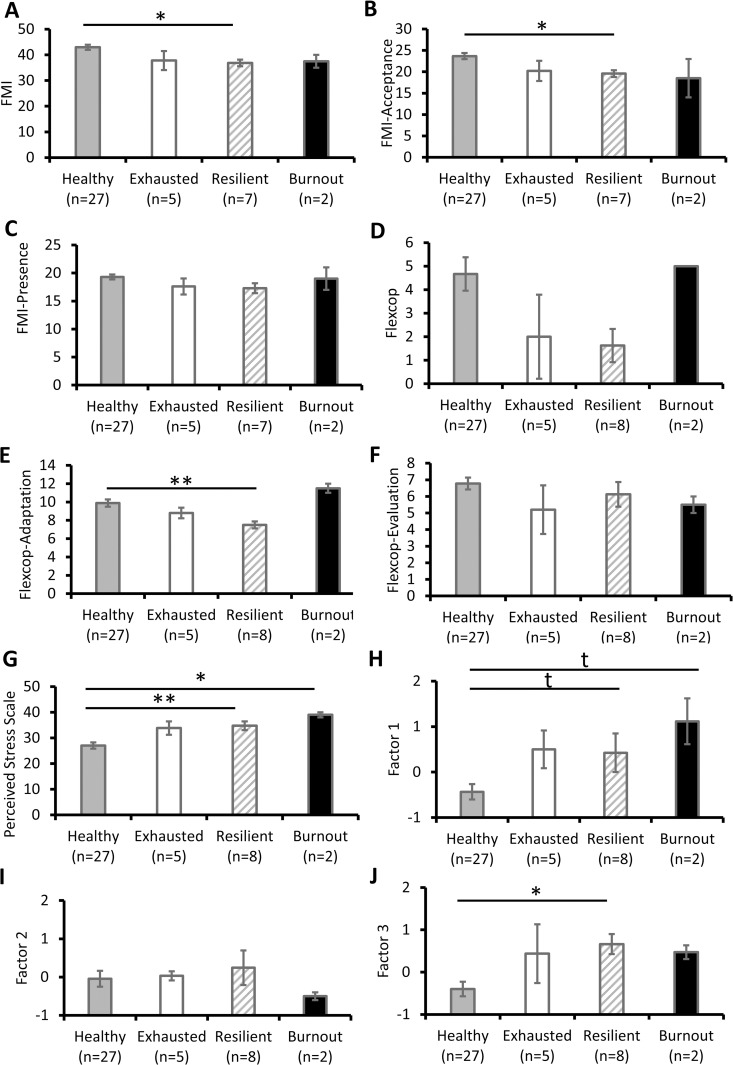
Psychological characterisation of participant groups. **A.** FMI scores. One participant in the Resilient group did not complete the FMI questionnaire. **B.** Acceptance dimension of FMI scores. **C.** Presence dimension of FMI scores. **D.** Coping flexibility scores. **E.** Adaptation dimension of coping flexibility scores. **F.** Evaluation dimension of coping flexibility scores. **G.** Perceived stress scale scores. **H.** Factor 1 of the initial factorial analysis. **I.** Factor 2 of the initial factorial analysis. **J.** Factor 3 of the initial factorial analysis. Results are expressed as mean ± SEM. t: p < 0.10, *: p < 0.05, **: p < 0.01; based on post-hoc tests. Flexcop, coping flexibility; FMI, Freiburg Mindfulness Inventory.

The psychological characterisation of each group on D0 is shown in Fig 3A–3J. All comparisons were made with healthy participants.

Resilient participants had lower FMI (F(3,37)=3.46, p = 0.026 η2 = 0.22; *post-hoc*, p = 0.032; Fig 3A), lower FMI-Acceptance (F(3,37)=3.50, p = 0.025 η2 = 0.22; *post-hoc*, p = 0.046; Fig 3B), and no difference in FMI-Presence (F(3,37)=1.65, p = 0.19 η2 = 0.12; Fig 3C). Resilient participants had lower Flexcop-Adaptation (F(3,37)=4.62, p = 0.008 η2 = 0.27; *post-hoc*, p = 0.007; Fig 3E), and no difference for the total Flexcop (F(3,38)=2.20, p = 0.104 η2 = 0.15; Fig 3D) or the Flexcop-Evaluation (F(3,38)=1.03, p = 0.39 η2 = 0.08; Fig 3F). Resilient participants had higher PSS (F(3,38)=6.35, p = 0.001 η2 = 0.33; *post-hoc*, p = 0.007; Fig 3G). Resilient participants had a tendency to higher Factor 1 of the initial factorial analysis (F(3,38)=3.79, p = 0.02 η2 = 0.24; *post-hoc*, p = 0.098; Fig 3H), no difference for Factor 2 (F(3,38)=0.31, p = 0.82 η2 = 0.02; Fig 3I), and higher Factor 3 (F(3,38)=3.20, p = 0.03 η2 = 0.21; *post-hoc*, p = 0.03; Fig 3J). Burnout participants had a tendency to higher Factor 1 (F(3,38)=3.79, p = 0.02 η2 = 0.24; *post-hoc*, p = 0.08; Fig 3H).

#### d. Subjective sleep characterisation.

**Fig 4:**
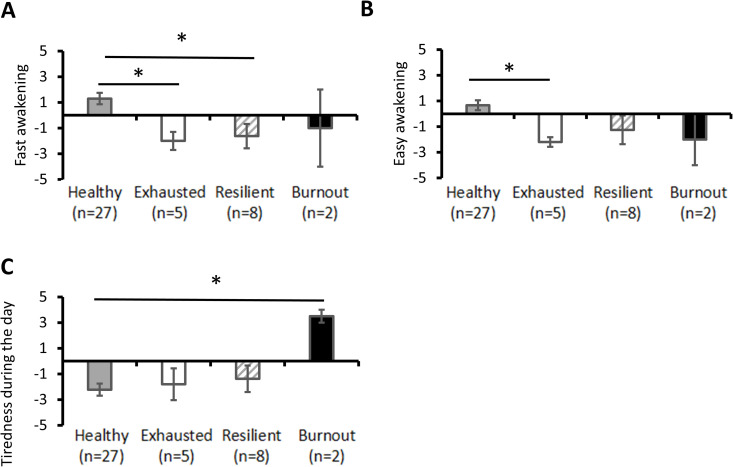
Psychological profiles in relation to sleep of participant groups. **A.** Fast awakening scores. **B.** Easy awakening scores. **C.** Tiredness during the day. Results are expressed as mean ± SEM. *: p < 0.05, **: p < 0.01; based on post-hoc tests.

The psychological profiles in relation to sleep of short-term burnout changes on D21 compared with D0 are shown in Fig 4. All comparisons were made with healthy participants.

Resilient participants had lower fast awakening (F(3,41)=5.01, p = 0.005 η2 = 0.28; *post-hoc*, p = 0.013; Fig 4A). Exhausted participants had lower fast awakening (F(3,41)=5.01, p = 0.005 η2 = 0.28; *post-hoc*, p = 0.02; Fig 4A) and lower easy awakening (F(3,41)=3.86, p = 0.02 η2 = 0.23; *post-hoc*, p = 0.03; Fig 4B). Burnout participants were more tired during the day (F(3,41)=3.21, p = 0.03 η2 = 0.20; *post-hoc*, p = 0.012; Fig 4C).

#### e. Electrophysiological characterisation.

**Fig 5:**
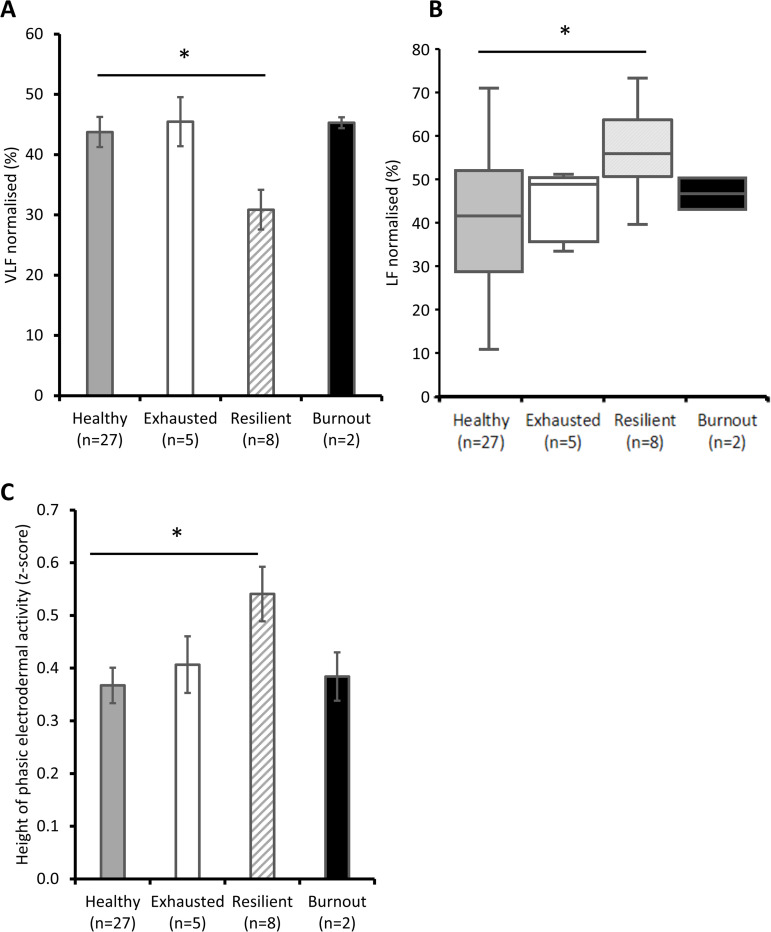
Electrophysiological characterisation of participant groups. The recordings were carried out during the emotional exposure protocol on D0. **A**. VLF normalised. Results are expressed as mean ± SEM. *: p < 0.05; based on post-hoc tests. VLF, very low frequencies. **B**. LF normalised. LF, low frequencies. The central line in the boxplot indicates the median, while bottom and top edges indicate 25th and 75th percentiles, respectively. Whiskers extend to the most extreme data points not considered outliers, and if present, outliers are plotted individually as circles. *, p < 0.05; based on post-hoc tests for Kruskal-Wallis test. **C.** Height of phasic electrodermal activity. Results are expressed as mean ± SEM. *: p < 0.05; based on post-hoc tests.

Electrophysiological parameters of the four groups are shown in Fig 5. All comparisons were made with healthy participants. Only significant results are reported.

Resilient participants had a lower normalised VLF (F(3,41)=2.72, p = 0.06 η2 = 0.18; *post-hoc*, p = 0.03; Fig 5A), a higher normalised LF (H(3,41)=7.95 p = 0.047, z = 2.78, p = 0.03; Fig 5B) and a higher height of phasic EDA (F(3,41)=2.34, p = 0.09 η2 = 0.16; *post-hoc*, p = 0.03; Fig 5C). No differences were observed for normalised HF (F(3,41)=0.83, p = 0.48 η2 = 0.06), the LF/HF ratio (F(3,41)=1.20, p = 0.32 η2 = 0.09), and the EDA tonic component (F(3,41)=0.69, p = 0.56 η2 = 0.05).

### 3. Relationship between D0 variables and D21 burnout

The correlation of D21 BMS to D0 variables is shown in S3 Table for the total population, and for the healthy population on D0 (healthy and exhausted groups).

For the total population, BMS on D21 positively correlated with Factor 1 (r = 0.40, r^2^ = 0.16, p = 0.01) and Factor 3 (r = 0.35, r^2^ = 0.12, p = 0.02). BMS on D21 negatively correlated with FMI on D0 (r=−0.40, r^2^ = 0.16, p = 0.009), the FMI-Acceptance on D0 (r=−0.41, r^2^ = 0.17, p = 0.007), and the Flexcop-Evaluation on D0 (r=−0.31, r^2^ = 0.10, p = 0.049) (S3 Table). BMS on D21 positively correlated with PSS on D0 (r = 0.53, r^2^ = 0.28, p = 0.0003; S3 Table).

Similar results were obtained in the healthy population on D0 (healthy and exhausted groups, n = 32; S3 Table), except no statistically significant correlations were shown for Flexcop-Evaluation.

## Discussion

The main results of this study are the characterisation of the four groups of burnout development and their physio-psychological markers. The four groups are “healthy,” “exhausted,” “resilient,” and “burnout.” Mindfulness scores at the end of deployment (D0) negatively correlated with BMS 21 days later. Conversely, PSS at the end of deployment (D0) positively correlated with BMS 21 days later.

### 1. Early stages of burnout development in caregivers

The caregiver population was studied in the specific context of a field deployment in a military intensive care hospital during the COVID-19 pandemic. Some participants had non-pathological depression (n = 1), anxiety (n = 4), PTSD (n = 3), and severe burnout (n = 1) on D0. To describe the short-term changes in burnout for caregivers working in ICUs in a pandemic context, we grouped participants in accordance with their burnout status on D0 and D21. The small sample size limits the conclusion.

Resilient participants had high BMS on D0, which normalised on D21, although their BMS improvement was modest (less than 10 points). Resilient participants were: under stress on D0, as suggested by high PSS; had high sympathetic activation, as suggested by high phasic EDA; and had low parasympathetic tone, as suggested by low VLF [35]. They also had subjective poor sleep, characterised by slower awakening. These results could be explained as a prolonged affective recovery from the stressful context of caregiving in an ICU during the COVID-19 pandemic. The stressed, but resilient participants had low FMI and Flexcop-Adaptation.

Conversely, exhausted participants had BMS that increased a lot from D0 to D21, reaching a BMS score compatible with burnout [25]. We therefore considered these participants as exhausted on D0. Interestingly, they did not have psychological abnormalities. Harder and slower awakenings suggested their strong need for sleep [36]. Need for sleep was greater in exhausted participants compared with resilient participants, suggesting that exhausted participants were in a more severe status than resilient participants. We wonder whether there is a step-by-step increase in adaptation under stress for resilience (high autonomic reactivity related to a defective control of emotion followed by sleep recovery) and exhaustion (enhanced heterostasis due to a regulated stress response). Nevertheless, exhausted participants remained well-adapted during the study, as exhaustion symptoms appeared after the end of deployment (D0).

Burnout participants did not present differences in FMI or Flexcop, in spite of increased perceived stress and major sleep disorders that altered the participants’ daily functioning. The very small group size (two participants) may explain this surprising result.

### 2. Association between mindfulness, coping flexibility, and burnout

On D0, burnout was associated with a low level of mindfulness, in agreement with another study [9]. We explored this observation further using a factorial analysis that showed the main factor (Factor 3) explaining burnout was associated with FMI and Flexcop. The role of mindfulness in the protection of the individual in the early phase of burnout onset deserves discussion. The negative correlation between D0 FMI and D21 BMS argues for mindfulness’s potential protective role against burnout. This is congruent with the negative association between mindfulness and risk for anxiety, depression, and burnout [37].

### 3. Limitations

The methodological limitations of this study relate to the low number of participants in each group, and the lack of control over participants’ activities from D0 to D21. These limitations reflect the environmental reality. The eight participants lost to follow-up on D21 also limit the study’s power. Nevertheless, half of those lost to follow-up were from the non-burnout group on D0, and the other half were from the burnout group on D0, suggesting loss to follow-up was not related to burnout. We did not control the amount of rest days each participant could take between D0 and D21. Nevertheless, at the end of deployment, all participants were able to return to their previous working environment, but in the complicated context of the COVID-19 pandemic, in which the French population was in lockdown. This lockdown, which exacerbates fatigue [38], is likely to have limited the variability that any rest days could have generated. Finally, as these data were obtained in the COVID-19 pandemic context within a specific population and context, our results have limited extrapolation to other environments and caregivers.

## Conclusion

This original study, carried out in a working environment, showed that some caregivers working in a military intensive care field hospital during the COVID-19 pandemic exhibited early stress and emotional dysregulation, which could constitute the initial steps toward burnout onset 21 days later. The subjective deterioration in sleep quality during a period with an exceptionally high workload appears to be a relevant clinical symptom that reflects the caregivers’ experiences. Mindfulness and, to a lesser extent, coping flexibility appear to be protective factors for the onset of burnout, while stress perception and physiological emotional regulation appear to be key markers of burnout onset. Future studies could clarify the extent to which these subjective measures can predict the onset of early burnout. Several studies warn about the need to prevent and manage burnout among intensive care healthcare workers [39,40]. We propose that interventions focusing on mindfulness and coping flexibility could potentially counteract early burnout in an ICU setting.

## Supporting information

S1 Table Number of participants on day 0 classified by anxiety, depression, and post-traumatic stress disorder scores in relation to burnout status. HADS, Hospital Anxiety and Depression Scale; PCL-5, PTSD Checklist for DSM-5; PTSD, post-traumatic stress disorder.
(PDF)

S2 Table Characteristics of participants on day 0. **A.** Sociodemographic and morphologic characteristics. **B.** Psychopathological categories among groups. Results are expressed as mean ± SEM for quantitative measures, and as number for qualitative measures. PTSD, post-traumatic stress disorder.
(PDF)

S3 Table Correlations between day 0 variables and day 21 burnout status. **A.** Total population. **B.** Healthy population on day 0. Results are expressed as Pearson correlation r values and their p-values. r^2^ higher or equal to 0.10 are in bold. BMS, Burnout Measure, Short Version; D, day; Flexcop, coping flexibility; FMI, Freiburg Mindfulness Inventory; ns, not significant; PSS, Perceived Stress Scale.
(PDF)

S1 Fig Psychopathological questionnaires in function of groups. **A.** HADS-Depression score on D0. **B.** HADS-Anxiety score on D0. **C.** PCL-5 score on D0. Results are expressed as mean ± SEM. HADS, Hospital Anxiety and Depression Scale; PCL-5, Post-Traumatic Stress Disorder Checklist for DSM-5. One participant in the Resilient group did not complete these questionnaires.
(PDF)

S1 File Supplementary materials. (PDF)
