# Supplementary material for: Influence of mindfulness and coping flexibility in the early phases of burnout development in intensive care unit healthcare workers during the COVID-19 pandemic
Source: PLoS One. 2025 Aug 21;20(8):e0328064. doi: 10.1371/journal.pone.0328064 (PMC12370081; doi:10.1371/journal.pone.0328064)
Supplement: S1 Table — HADS, Hospital Anxiety and Depression Scale; PCL-5, PTSD Checklist for DSM-5; PTSD, post-traumatic stress disorder. (PDF) [file pone.0328064.s001.pdf]

Supplementary Table 1: Number of participants on day 0 classified by anxiety, depression, and post-traumatic stress disorder scores in relation to burnout status. HADS, Hospital Anxiety and Depression Scale; PCL-5, PTSD Checklist for DSM-5; PTSD, post-traumatic stress disorder.

	Non-Burnout	Burnout	Statistics
Anxious/Non-anxious (HADS-Anxiety)	3/32	1/12	χ^2 p=0.92
Depression/Non-depression (HADS-Depression)	0/35	1/12	χ^2 p=0.097
PTSD/Non-PTSD (PCL-5)	2/34	1/12	χ^2 p=0.78
