# Supplementary material for: Influence of mindfulness and coping flexibility in the early phases of burnout development in intensive care unit healthcare workers during the COVID-19 pandemic
Source: PLoS One. 2025 Aug 21;20(8):e0328064. doi: 10.1371/journal.pone.0328064 (PMC12370081; doi:10.1371/journal.pone.0328064)
Supplement: S2 Table — A. Sociodemographic and morphologic characteristics. B. Psychopathological categories among groups. Results are expressed as mean ± SEM for quantitative measures, and as number for qualitative measures. PTSD, post-traumatic stress disorder. (PDF) [file pone.0328064.s002.pdf]

Supplementary Table 2: Characteristics of participants on day 0. A. Sociodemographic and morphologic characteristics. **B.** Psychopathological categories among groups. Results are expressed as mean ± SEM for quantitative measures, and as number for qualitative measures. PTSD, post-traumatic stress disorder.

A

	Healthy	Exhausted	Resilient	Burnout	Statistics
Age (year)	34.6 ± 2.5	33.8 ± 3.9	35.9 ± 2.1	32.5 ± 0.5	$F(3,41)=0.07$ $p=0.98$
Gender (Women/Men)	12/15	2/3	5/3	1/1	X^2: $Q_{obs}=0.94$ $p=0.82$
Weight (kg)	71.5 ± 3.6	66.2 ± 3.9	61.5 ± 2.0	80.0 ± 15.0	$F(3,41)=1.2$ $p=0.34$
Height (cm)	173.2 ± 2.1	173.8 ± 4.3	170.6 ± 2.1	180.5 ± 2.5	$F(3,41)=0.6$ $p=0.6$
Number of deployments (count)	6.8 ± 1.1	2.8 ± 1.1	6.1 ± 1.5	2.0 ± 0.0	$F(3,41)=1.3$ $p=0.3$

B

	Healthy	Exhausted	Resilient	Burnout	Statistics
Anxious /Non-anxious	1/26	1/4	0/8	0/2	X^2: $Q_{obs}=3.13$ p=0.37
Depression /Non-depression	0/27	0/5	1/7	0/2	X^2: $Q_{obs}=4.35$ p=0.23
PTSD/Non-PTSD	1/26	1/4	1/7	0/2	X^2: $Q_{obs}=2.23$ p=0.53
