# Supplementary material for: Influence of mindfulness and coping flexibility in the early phases of burnout development in intensive care unit healthcare workers during the COVID-19 pandemic
Source: PLoS One. 2025 Aug 21;20(8):e0328064. doi: 10.1371/journal.pone.0328064 (PMC12370081; doi:10.1371/journal.pone.0328064)
Supplement: S3 Table — A. Total population. B. Healthy population on day 0. Results are expressed as Pearson correlation r values and their p-values. r2 higher or equal to 0.10 are in bold. BMS, Burnout Measure, Short Version; D, day; Flexcop, coping flexibility; FMI, Freiburg Mindfulness Inventory; ns, not significant; PSS, Perceived Stress Scale. (PDF) [file pone.0328064.s003.pdf]

A

Total population	BMS D21
FMI	**r=–0.40 r^2=0.16 p=0.009**
FMI-Presence	r=–0.25 r^2=0.06 p=0.12
FMI-Acceptance	**r=–0.41 r^2=0.17 p=0.007**
Flexcop	r=–0.19 r^2=0.04 p=0.22
Flexcop-Evaluation	**r=–0.31 r^2=0.10 p=0.049**
Flexcop-Adaptation	r=–0.03 r^2=0.0009 p=0.86
PSS	**r=0.53 r^2=0.28 p=0.0003**

B

Healthy population at D0 (healthy and exhausted groups; n=32)	BMS D21
FMI	**r=–0.41 r^2=0.17 p=0.02**
FMI-Presence	r=–0.28 r^2=0.08 p=0.12
FMI-Acceptance	**r=–0.42 r^2=0.18 p=0.02**
Flexcop	r=–0.24 r^2=0.06 p=0.18
Flexcop-Evaluation	r=–0.28 r^2=0.08 p=0.11
Flexcop-Adaptation	r=–0.16 r^2=0.03 p=0.38
PSS	**r=0.51 r^2=0.26 p=0.003**

Supplementary Table 3: Correlations between day 0 variables and day 21 burnout status. A. Total population. **B.** Healthy population on day 0. Results are expressed as Pearson correlation r values and their p-values. r^2 higher or equal to 0.10 are in bold. BMS, Burnout Measure, Short Version; D, day; Flexcop, coping flexibility; FMI, Freiburg Mindfulness Inventory; ns, not significant; PSS, Perceived Stress Scale.
