# Supplementary material for: Influence of mindfulness and coping flexibility in the early phases of burnout development in intensive care unit healthcare workers during the COVID-19 pandemic
Source: PLoS One. 2025 Aug 21;20(8):e0328064. doi: 10.1371/journal.pone.0328064 (PMC12370081; doi:10.1371/journal.pone.0328064)
Supplement: S1 Fig — A. HADS-Depression score on D0. B. HADS-Anxiety score on D0. C. PCL-5 score on D0. Results are expressed as mean ± SEM. HADS, Hospital Anxiety and Depression Scale; PCL-5, Post-Traumatic Stress Disorder Checklist for DSM-5. One participant in the Resilient group did not complete these questionnaires. (PDF) [file pone.0328064.s004.pdf]

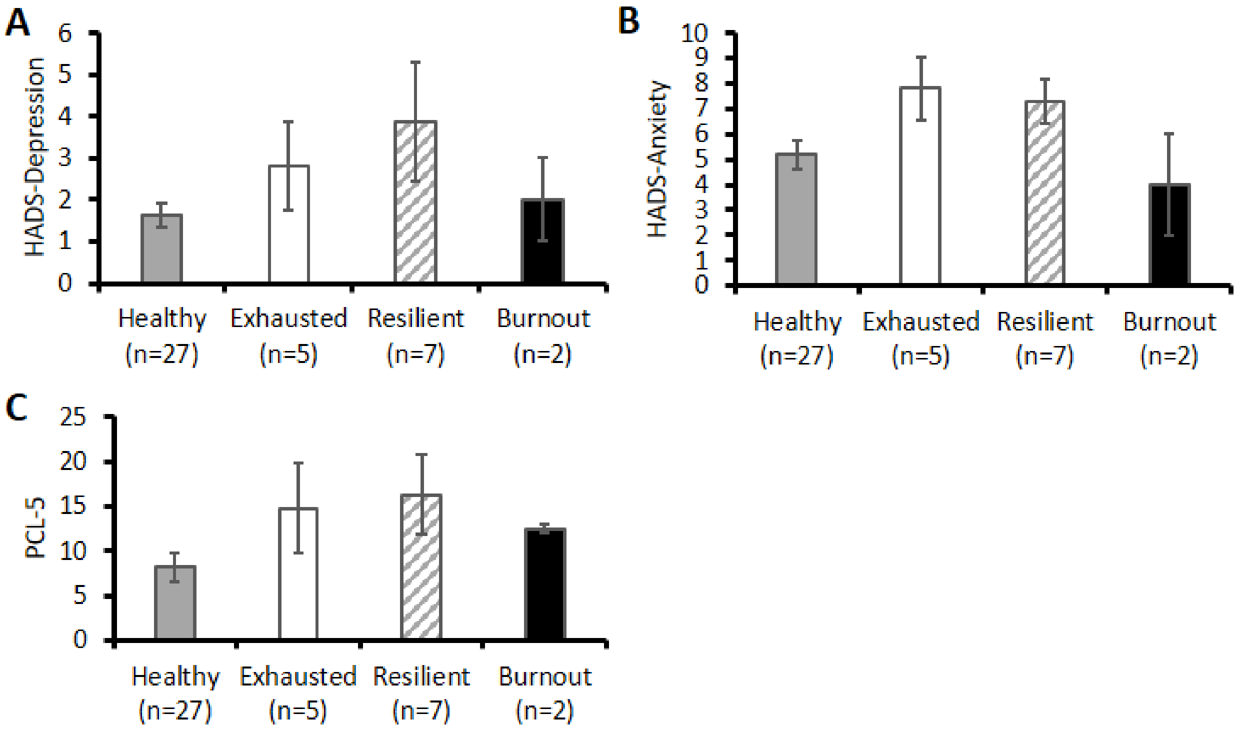

Supplementary Figure 1: Psychopathological questionnaires in function of groups. A. HADS-Depression score on D0. **B.** HADS-Anxiety score on D0. **C.** PCL-5 score on D0. Results are expressed as mean ± SEM. HADS, Hospital Anxiety and Depression Scale; PCL-5, Post-Traumatic Stress Disorder Checklist for DSM-5. One participant in the Resilient group did not complete these questionnaires.
