# Supplementary material for: Influence of mindfulness and coping flexibility in the early phases of burnout development in intensive care unit healthcare workers during the COVID-19 pandemic
Source: PLoS One. 2025 Aug 21;20(8):e0328064. doi: 10.1371/journal.pone.0328064 (PMC12370081; doi:10.1371/journal.pone.0328064)
Supplement: S1 File — (PDF) [file pone.0328064.s005.pdf]

Supplementary Materials

Sociodemographic and morphometric assessment

Sociodemographic variables were age, gender, number of deployments. Morphologic variables were weight and height. A summary of these data is presented in Supplementary Table 2.

Psychopathological evaluations

Cronbach alpha coefficients ranged from acceptable to good for all data from the psychological and pathological questionnaires. Questionnaires were excluded from analysis when more than two items were not completed. When only one item was not completed, the mean of the data from the remaining questions was calculated.

Electrophysiological recordings

ECG data were recorded using sensors placed on the chest in accordance with the standard Einthoven DII derivation. EDA was recorded by placing an electrode on the last phalanx of each of the index and middle fingers of the non-dominant hand. The signal was acquired by BioNomadix ECG2-R and PPGED-R amplifier modules connected to a BIOPAC MP160 (BIOPAC Systems, Inc., Goleta, CA, USA) managed by AcqKnowledge version 5.0 software (BIOPAC Systems, Inc.) at a sampling frequency of 1000 Hz.

Physiological signal preprocessing

Kubios HRV Premium software version 4.0.1 (Kubios Oy, Kuopio, Finland) was used to automatically detect R-peaks from the ECG data. The results were visually checked. The resulting RR intervals were calculated and used for heart rate variability (HRV) analysis.

The processing of the tonic and phasic EDAs was carried out using MATLAB software R2019a (MathWorks, Natick, MA, USA). The EDA signal was z-score normalised and then separated into its phasic and tonic components by a validated algorithm (Greco, Valenza, Lanata, Scilingo, & Citi, 2016).

Physiological signal analyses

Heart rate variability analysis

HRV analysis using the RR interval time series was carried out using MATLAB software. The temporal analysis was based on the RMSSD, a parasympathetic activity index (Electrophysiology, 1996). RR intervals were interpolated to a 2-Hz RR time series that was detrended before analysis by fast Fourier transformation (Welch method with a moving window and an overlap of 50%) to calculate the spectral component of HRV parametric analyses—VLF (0.0033-0.04 Hz), LF (0.04-0.15 Hz), and HF (0.15-0.5 Hz)—which were also expressed relatively as VLF+LF+HF as a percentage. The LF/HF ratio was also calculated.

Electrodermal activity analysis

EDA was analysed by determining its tonic component, representing basal sympathetic activity (i.e., the equilibrium between sympathetic and parasympathetic activity), and the phasic activity, representing bursts of sympathetic activity (Posada-Quintero & Chon, 2020). The number and the height of peaks of the phasic EDA component was determined by the integrated MATLAB function *findpeaks* using a threshold of 0.1 standard deviations of the mean.

Bibliography

Electrophysiology, T. F. o. T. E. S. o. C. a. T. N. A. S. o. P. a. (1996). Heart rate variability: standards of measurement, physiological interpretation and clinical use. Task Force of the European Society of Cardiology and the North American Society of Pacing and Electrophysiology. *Circulation, 93*(5), 1043-1065. Retrieved from http://www.ncbi.nlm.nih.gov/pubmed/8598068

Greco, A., Valenza, G., Lanata, A., Scilingo, E. P., & Citi, L. (2016). cvxEDA: A Convex Optimization Approach to Electrodermal Activity Processing. *IEEE Trans. Biomed. Eng., 63*(4), 797-804. doi:10.1109/TBME.2015.2474131

Posada-Quintero, H. F., & Chon, K. H. (2020). Innovations in Electrodermal Activity Data Collection and Signal Processing: A Systematic Review. *Sensors (Basel), 20*(2). doi:10.3390/s20020479
